# Nitrogen addition alters soil fungal communities, but root fungal communities are resistant to change

**DOI:** 10.3389/fmicb.2022.1033631

**Published:** 2023-01-25

**Authors:** Alyssa A. Carrell, Brittany B. Hicks, Emilie Sidelinger, Eric R. Johnston, Sara S. Jawdy, Miranda M. Clark, Dawn M. Klingeman, Melissa A. Cregger

**Affiliations:** ^1^Biosciences Division, Oak Ridge National Laboratory, Oak Ridge, TN, United States; ^2^Department of Civil and Environmental Engineering, University of Michigan, Ann Arbor, MI, United States; ^3^Department of Environmental Engineering and Earth Sciences, Clemson University, Clemson, SC, United States

**Keywords:** mycorrhizal fungi, nitrogen addition, plant-microbe interactions, *Populus*, symbiosis

## Abstract

Plants are colonized by numerous microorganisms serving important symbiotic functions that are vital to plant growth and success. Understanding and harnessing these interactions will be useful in both managed and natural ecosystems faced with global change, but it is still unclear how variation in environmental conditions and soils influence the trajectory of these interactions. In this study, we examine how nitrogen addition alters plant-fungal interactions within two species of *Populus* - *Populus deltoides* and *P*. *trichocarpa*. In this experiment, we manipulated plant host, starting soil (native vs. away for each tree species), and nitrogen addition in a fully factorial replicated design. After ~10 weeks of growth, we destructively harvested the plants and characterized plant growth factors and the soil and root endosphere fungal communities using targeted amplicon sequencing of the ITS2 gene region. Overall, we found nitrogen addition altered plant growth factors, e.g., plant height, chlorophyll density, and plant N content. Interestingly, nitrogen addition resulted in a lower fungal alpha diversity in soils but not plant roots. Further, there was an interactive effect of tree species, soil origin, and nitrogen addition on soil fungal community composition. Starting soils collected from Oregon and West Virginia were dominated by the ectomycorrhizal fungi *Inocybe* (55.8% relative abundance), but interestingly when *P*. *deltoides* was grown in its native West Virginia soil, the roots selected for a high abundance of the arbuscular mycorrhizal fungi, *Rhizophagus*. These results highlight the importance of soil origin and plant species on establishing plant-fungal interactions.

## 1. Introduction

Over the last century, increases in atmospheric nitrogen (N) concentrations have resulted in significant N deposition within soils across the globe (Canfield et al., 2010). Increased levels of this limiting nutrient within soils result in significant changes in ecosystem structure and function. Belowground, N addition has significant effects on soil microbial diversity, biomass, and growth (Zhang et al., 2018). Root associated microbial communities contain up to 10^11^ microbial cells per gram plant root and are primarily comprised of bacteria and fungi (White et al., 2017). These communities regulate organic matter decomposition, nutrient cycling, and are key players in plant health and growth, thus changes in these communities may significantly impact plant physiology and ecosystem function.

Soil fungi (the soil mycobiome) are vital members in belowground microbial communities. These fungi include prevalent decomposers and beneficial plant symbionts. Interactions between plants and fungi belowground are essential components for plant health, growth, and response to abiotic stress (Hacquard and Schadt, 2015; Vandenkoornhuyse et al., 2015; Cregger et al., 2021). Understanding factors that regulate and alter these interactions is imperative if we aim to leverage these interactions to develop sustainable ecosystems and influence the way in which carbon and nutrients are cycled and stored within soils (de Vries et al., 2018). Because many plant processes rely on or are improved by associations with soil fungi, it has become increasingly important to study the plant and its microbiota as a joint system (Hacquard and Schadt, 2015).

Within the myriad of possible plant-fungal interactions occurring, plant-mycorrhizal associations are widespread with the two most common mycorrhizal types being ectomycorrhizae (ECM) and arbuscular mycorrhizae (AM). Belowground plant interactions with these fungi have been shown to increase water uptake and nutrient acquisition, and shift soil carbon storage (Gehring and Connell, 2006; Bonfante and Genre, 2010; Miransari, 2011; Liu et al., 2015). Numerous studies have highlighted that global change factors influence mycorrhizal communities (Compant et al., 2010; Bellgard and Williams, 2011; Pickles et al., 2012; Bennett and Classen, 2020; Yang et al., 2021), but it is unclear how variation in the diversity and abundance of these communities alter plant host traits. Most plants associate with one type of mycorrhizal fungi, and individual plant species are less likely to associate with both AM and ECM fungal species (Teste et al., 2020). Contrary to this, trees within the genus *Populus* have the unique feature that they associate with both AM and ECM simultaneously in natural settings (Gehring and Connell, 2006; Cregger et al., 2018).

*Populus* has become a model tree species for studying plant-microbe interactions due to its fast growth, clonal propagation, and vast genomic resources (Cregger et al., 2021). *Populus* species are globally distributed and are often keystone members within forested ecosystems (Suarez-Gonzalez et al., 2016; Kivinen et al., 2020). *Populus* is commonly found in riparian environments at the interface between terrestrial and aquatic ecosystems, serving a unique role within these environments (Cronk, 2005). Further, *Populus* is economically important to industries such as lumber, paper, and potentially cellulose-derived biofuels (Sannigrahi et al., 2010; Happs et al., 2021), largely due to their fast growth rate and ability to be grown on marginal lands.

Previous research has demonstrated that fungal communities differ between *Populus* species and genotypes, and *Populus* clones have varying degrees of AM and ECM colonization (Gehring and Connell, 2006; Martin et al., 2008; Bonito et al., 2014; Cregger et al., 2018). Plant genetic factors, starting soil inoculum, and abiotic variables such as soil moisture influence fungal community diversity and composition (Shakya et al., 2013; Veach et al., 2020) as well as rates of AM vs. ECM colonization and abundance on *Populus* (Gehring and Connell, 2006). Interestingly, when grown in a common environment and exposed to variation in soil nitrogen content, both ECM and AM colonization and diversity decreases with increasing levels of soil N (Hodge and Storer, 2015; Yang et al., 2021), but it is unclear if this pattern is consistent across *Populus* species. Further it is unclear how soil N concentration alters broader *Populus*-fungal interactions across *Populus* species, and if changes in these interactions are contingent upon soil origin.

The overarching goal of this study was to determine the impact of increased soil N concentration on belowground plant-fungal interactions within two *Populus* species, and to identify if soil origin altered the impact of N on *Populus*-fungal interactions. Specifically, we ask: (1) Does N addition result in convergence of the mycobiome across two *Populus* species and across two soil types with distinct initial microbial communities, (2) Does N addition alter core fungal taxa present across two *Populus* species, (3) Does N addition change plant-mycorrhizal associations consistently within two *Populus* species, and finally (4) Do changes in the fungal community correlate with alterations in plant traits? We hypothesize N addition will result in convergence of the mycobiome within roots and soils regardless of plant species or soil origin, and N addition will result in a decoupling of beneficial *Populus*-fungal interactions. Specifically, we hypothesize that N addition will result in decreased diversity and relative abundance of both AM and ECM in plant roots and plant associated soils.

## 2. Materials and methods

### 2.1. Experimental design

**Figure 1:**
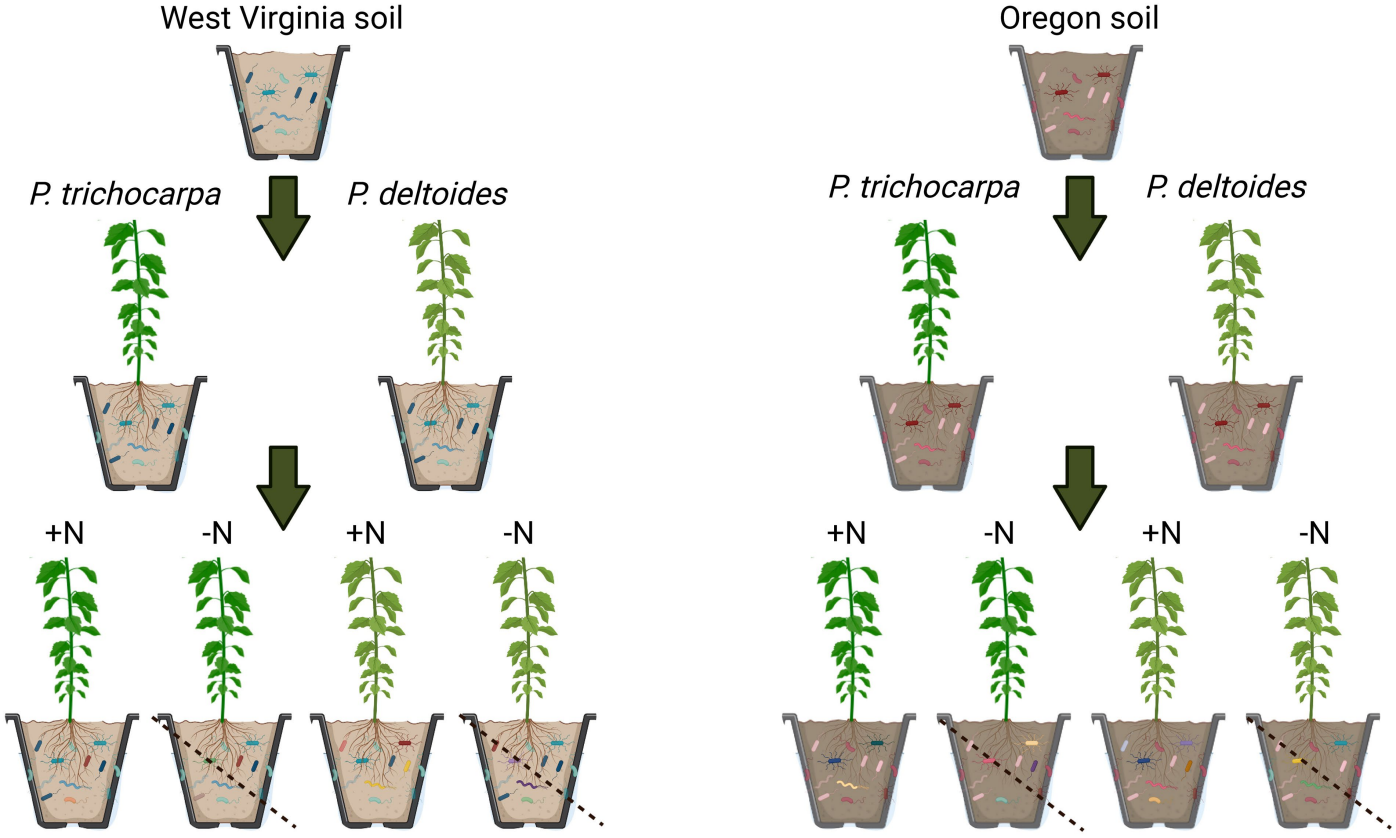
Experimental design. Cuttings of *P*. *trichocarpa* and *P*. *deltoides* and their accompanying field soils were obtained from Oregon and West Virginia. A total of 160 cuttings (80 *P*. *deltoides*/80 *P*. *trichocarpa*) were propagated and 40 cuttings from each species (*P*. *trichocarpa*/*P*. *deltoides*) were grown in each soil mixture (Oregon/West Virginia) for ~10 weeks and half of the plants received Hoagland’s fertilizer with nitrogen, while the other half received Hoagland’s fertilizer without nitrogen (referred to as N+ and N− treatments, respectively).

Cuttings of *P*. *trichocarpa* (genotype GW9791*)* and *P*. *deltoides* (genotype WV94; Coyle et al., 2006) and their accompanying field soils were obtained from existing common garden experiments located in Oregon and West Virginia where these *Populus* species are each native. Three soil subsamples were collected from Oregon and West Virginia for initial chemical and mycobiome analyses as described below. A total of 160 cuttings (80 *P*. *deltoides*/80 *P*. *trichocarpa*) were propagated on March 28, 2019 in autoclaved potting mix in a greenhouse at Oak Ridge National Laboratory (Oak Ridge, TN) to establish growth. Prior to onset of experimental treatments, field-collected soils from Oregon and West Virginia were mixed with sterile sand (50/50 mixture) to induce N-limiting conditions. Forty cuttings from each species (*P*. *trichocarpa*/*P*. *deltoides*) were grown in each soil mixture (Oregon/West Virginia) for ~10 weeks in 1.6-liter tree pots (Figure 1). During the experiment, Hoagland’s fertilizer (Basal mix #2, Caisson Labs, Smithfield, UT) was applied weekly in 400 mL increments. Half of the plants received Hoagland’s fertilizer with N, while the other half received Hoagland’s fertilizer without N (henceforth referred to as N+ and N− treatments, respectively).

### 2.2. Plant and soil characterization

We assessed plant characteristics (number of leaves, plant height, stem diameter at two locations, and leaf chlorophyll content) upon the onset of the experiment in May and in July (~ 8 weeks into the experiment). Leaf chlorophyll content was measured using a SPAD-502 Meter (Spectrum Technologies, Inc., Aurora, IL, United States). For SPAD measurements, three leaves per plant were measured and the mean SPAD content was calculated per plant as performed previously (Veach et al., 2019). Plants were destructively harvested when they became root bound (root production was evident at the bottom of the pot), which differed slightly between N+ and N− by 2 weeks (late July for N+ and mid-August for N−), due to differences in growth rates between groups. Harvesting plants based on when they became root bound allow assessments to be made when plants were at similar physiological states. At harvest, a subset of leaves, stems, and roots were removed to assess percent plant carbon (C) and N. A second subset of roots were collected for ITS2 amplicon sequencing (see section “Root and soil microbiome characterization” below). Soil was homogenized during the destructive harvest and a subset was collected in a 50 mL sterile tube, flash frozen in liquid N_2_ and stored at −80°C until further processing. Given roots occurred continuously throughout the pot at harvest, soil was not partitioned into bulk soil/rhizosphere samples and this compartment will be referred to as ‘soil’ throughout the manuscript.

Initial characterization of field soils collected from Oregon and West Virginia was performed at the University of Georgia Agricultural and Environmental Services Laboratories - Soil, Plant, and Water Laboratory (Athens, GA, United States). For triplicate field soil subsamples, soil extractable P, K, Ca, Mg, Mn, and Zn were extracted using the Mehlich-1 method and measured using an inductively coupled plasma spectrograph (Supplementary Table S1). At the completion of the experiment, we assessed soil pH (LabFit AS-3000 pH Analyzer), NH_4_-N, and NO_3_-N (the phenate method for NH_4_^+^ and the cadmium reduction method for NO_3_^−^ (Robertson et al., 1999) within 10 replicate pots per experimental group. Additionally, we assessed %C and %N for stem, leaf, and root subsamples across the same 10 replicate trees using the Dumas Method (Kirsten and Hesselius, 1983). In brief, a representative sample (~0.5 g) was loaded into a ceramic sample boat and combusted in an oxygen atmosphere at 1,350°C in a Elementar Vario Max Total Combustion Analyzer. Elemental C and N were converted into CO_2_, NO_x_, and N_2_. These gases were then passed through the IR (infrared) cells to determine the carbon content and a TC (thermal conductivity) cell to determine N_2_. Results are reported as a ratio between the two percentages.

### 2.3. Root and soil microbiome characterization

Roots from five replicates across each experimental group (randomly chosen from the same subset of 10 that were used for plant and soil characterization above), were surface sterilized with three 30 s water wash cycles, followed by a 30 s wash of 70% ethanol, and another set of three 30 s water wash cycles. Fine roots were then dissected with a razor blade, pulverized in liquid N_2_ frozen blocks with the Qiagen TissueLyser at 30 rpm for 3 min with a 5-mm steel bead and DNA isolated with the Qiagen DNeasy PowerPlant Pro Kit (Qiagen, Venlo, Netherlands). To remove possible PCR inhibitors and ethanol carry over, DNA was purified with the Qiagen DNA cleanup kit and Agencourt AMPure XP beads (Beckman Coulter, Brea, CA, United States).

Five replicates from each experimental group, as well as triplicate subsamples from each of the preserved field soils, were used for soil DNA extraction. Soil DNA was extracted with the Qiagen DNeasy PowerSoil Pro Kit, using a Precellys 24 (Bertin Instruments, France) for lysis. For each sample, duplicate extractions of 0.25 g were performed. Duplicate extracts were quantified individually using a Qubit fluorometer and Qubit dsDNA Broad Range Assay Kit (Life Technologies, Carlsbad, CA, United States) and then combined. DNA extracts were purified with the DNeasy PowerClean Pro Cleanup Kit followed by Agencourt AMPure XP beads following the standard DNA cleanup protocol.

### 2.4. Amplicon library preparation

Amplicons were prepared following the 16S Metagenomic Sequencing Library preparation protocol (Part# 15044223 RevB, Illumina Inc. Hayward CA). Amplicons were amplified by polymerase chain reaction (PCR) with a mixture of custom ITS2 gene region primers for fungi designed to increase phylogenetic coverage within amplicon libraries (Dove et al., 2021), and then uniquely barcoded in a subsequent PCR with Illumina Nextera XT v2 indexes. Amplicon PCR contained 12.5 μL of 2x KAPA HiFi HotStart Ready Mix, 5 μM each of amplicon specific primers, 25–50 ng of template DNA, and water to a final reaction volume of 25 μL. Amplification parameters for amplicon PCR included initial denaturation at 95°C for 3 min, followed by 25 cycles of 95°C for 30 s, 55°C for 30 s, 72°C for 30 s, and a final extension at 72°C for 5 min. Following the initial amplification using target specific primers, 2 μL of the PCR reaction was visualized by gel electrophoresis (1% agarose). The remainder of the PCR reaction was cleaned with AMPure beads using a 0.8x ratio of beads to PCR reaction volume (23 μL) and resuspended in 50 μL of 10 mM Tris pH 8.5. To uniquely tag each amplicon, a second round of PCR (Index PCR) was performed in a 50 μL reaction volume with 25 μL of 2x KAPA HiFi HotStart Ready Mix, 5 μL of Nextera XT Index primer 1 (N7xx), 5 μL of Nextera XT index primer 2 (S5xx), 5 μL of cleaned DNA from the amplicon amplification and 10 μL water. Index PCR amplification parameters included initial denaturation at 95°C for 3 min, followed by 8 cycles of 95°C for 30 s, 55°C for 30 s, 72°C for 30 s, and a final extension at 72°C for 5 min. After the Index PCR, the amplified products were cleaned using AMPure beads (1x volume ratio) and resuspended in 25 μL of 10 mM Tris pH 8.5. The cleaned amplicons were quantified on a NanoDrop 1,000 (Thermo Fisher Scientific, Wilmington, DE, United States), pooled equimolarly, and validated on an Agilent Bioanalyzer (Agilent, Santa Clara, CA, United States) using a DNA7500 chip. The final pool was again AMPure bead purified (0.7× volume ratio) to remove any remaining small DNA fragments. Lastly, the sample concentration was quantified on a Qubit fluorometer with the broad range dsDNA assay (Life Technologies). Libraries were prepared for sequencing following the Illumina MiSeq denature and dilute libraries guide. PhiX control DNA was included in the sequencing run due to low base diversity associated with amplicon samples. Libraries were loaded into the sequencing cassette (v2 chemistry) and a paired end (2×251×8×8) sequencing was completed on an Illumina MiSeq Instrument (Illumina, San Diego, CA, United States).

### 2.5. Sequence data processing

Fungal sequences were processed with the QIIME 2 v 2019.10 platform (Bolyen et al., 2019). Paired sequences were demultiplexed with the plugin demux and quality filtered (denoised, dereplicated, chimera filtered, and pair-end merged) and processed into Sequence Variants (SVs) with the dada2 plugin (Callahan et al., 2016). Taxonomy was assigned using a pre-trained Naive Bayes classifier based on the Unite (ITS) database (Nilsson et al., 2019) and mitochondria, chloroplast and unassigned sequences were removed, and samples were rarefied to 12,000 reads. SVs were classified into functional guild using the FUNguild database (Nguyen et al., 2016), filtered to include only assignments greater than “probable” and subset to include AM and ECM fungi.

### 2.6. Statistical approaches

All statistical analyses were performed in R version 4.2.0 and visualized with ggplot2. For alpha diversity, Hill numbers (or effective numbers of species) were calculated on the full dataset with the package hillR (Chao et al., 2014) to control the contribution of rare taxa to the diversity metrics. In general, the diversity measures are weighted differently by the diversity order (^q^D), which calculates Hill numbers weighted differently by species abundance distributions (Hill, 1973; Chao et al., 2012). For example, at q = 0, all species are equally weighted, at q = 1, species are proportionally weighted to relative abundance, and at q = 2, rare species are down weighted. A three-way type II ANOVA was performed with the “car” package to test the effect of soil origin, tree species, and/or N addition on Hill numbers for overall fungal communities. Additionally, we performed an ANOVA and Tukey’s honest significant difference (HSD) *post hoc* analyses on the AM and ECM portion of the fungal communities. Beta diversity was visualized with non-metric multidimensional scaling (NMDS) based on Bray-Curtis distances. A PERMANOVA was performed to test the role of soil origin, tree species, and/or N addition on overall fungal community structure as well as AM and ECM fungal communities. We defined core taxa within the total mycobiome that uniquely occurred within soils, roots, *P*. *trichocarpa*, and *P*. *deltoides* at the genus level with the core_members (Shetty and Lahti, 2019) function from the *microbiome* package in R. We defined core taxa has having a prevalence of 95% with a relative abundance >0.001 in each considered dataset.

The relationship of plant and soil characteristics and tree species, soil origin, and N treatment was assessed with general linear models (GLM). Plant and soil characteristic correlations with microbiome taxa were assessed with the Pearson correlation test, with *p* values corrected for multiple comparisons by the false-discovery rate method. Statistical significance for all analyses was determined using *p*-value cutoff ≤0.05.

## 3. Results

### 3.1. Variation in fungal community alpha and beta diversity

The addition of N impacted microbial alpha and beta diversity differently in soil compared to roots. There was an interactive effect of N addition, tree species, and soil origin on soil fungal alpha diversity at q1 (Shannon diversity; *p* < 0.05) and q2 (Simpson diversity; *p* < 0.05). Contrary to this, there were no interactive effects of N addition, tree species, and soil origin on root fungal alpha diversity. There were significant main effects of soil origin, tree species and N addition on soil alpha diversity (Table 1). For example, on average, N addition decreased fungal alpha diversity in soils at q1 and q2 (*p* < 0.05) but did not alter alpha diversity in plant roots at any level of q (*p* > 0.05). Soil origin was the only predictor of root fungal community alpha diversity at q0 (*p* < 0.05).

**Table 1:** Alpha diversity results from analysis of variance tests of the relationship between Hill numbers (q = 0, q = 1, q = 2) and nitrogen addition, tree species, and soil origin.

Effects (df)	q = 0		q = 1		q = 2		*F*	*P*	*F*	*P*	*F*	*P*
	*Soil*	
Nitrogen addition (1)	0.5	0.62	14.8	**<0.001**	9.4	**0.004**
Species (1)	4.1	0.07	42.3	**<0.001**	33.2	**<0.001**
Soil origin (1)	57.0	**<0.001**	75.5	**<0.001**	27.0	**<0.001**
Species: nitrogen addition (1)	3.6	**0.04**	37.6	**<0.001**	22.4	**<0.001**
Soil origin: nitrogen addition (1)	1.7	0.2	18.4	**<0.001**	10.7	**0.002**
Species: soil origin (1)	0.4	0.61	20.0	**<0.001**	9.1	**0.005**
Species: soil origin: nitrogen addition (1)	1.9	0.17	30.3	**<0.001**	18.1	0.000
	*Root*	
Nitrogen addition (1)	0.8181	0.22	1.1683	0.27	2.1368	0.42
Species (1)	0.1986	0.66	0.0271	0.95	0.2703	0.7
Soil origin (1)	6.5342	**<0.001**	0.1488	0.1	0.2276	0.12
Species: nitrogen addition (1)	0.174	0.85	0.021	0.92	0.0861	0.72
Soil origin: nitrogen addition (1)	0.3519	0.21	2.8789	0.37	3.9624	0.21
Species: soil origin (1)	0.9654	0.14	0.8442	0.34	1.7907	0.14
Species: soil origin: nitrogen addition (1)	0.9157	0.15	3.1742	0.15	4.4897	0.06

Significant values (*p* < 0.05) are bolded.

**Figure 2:**
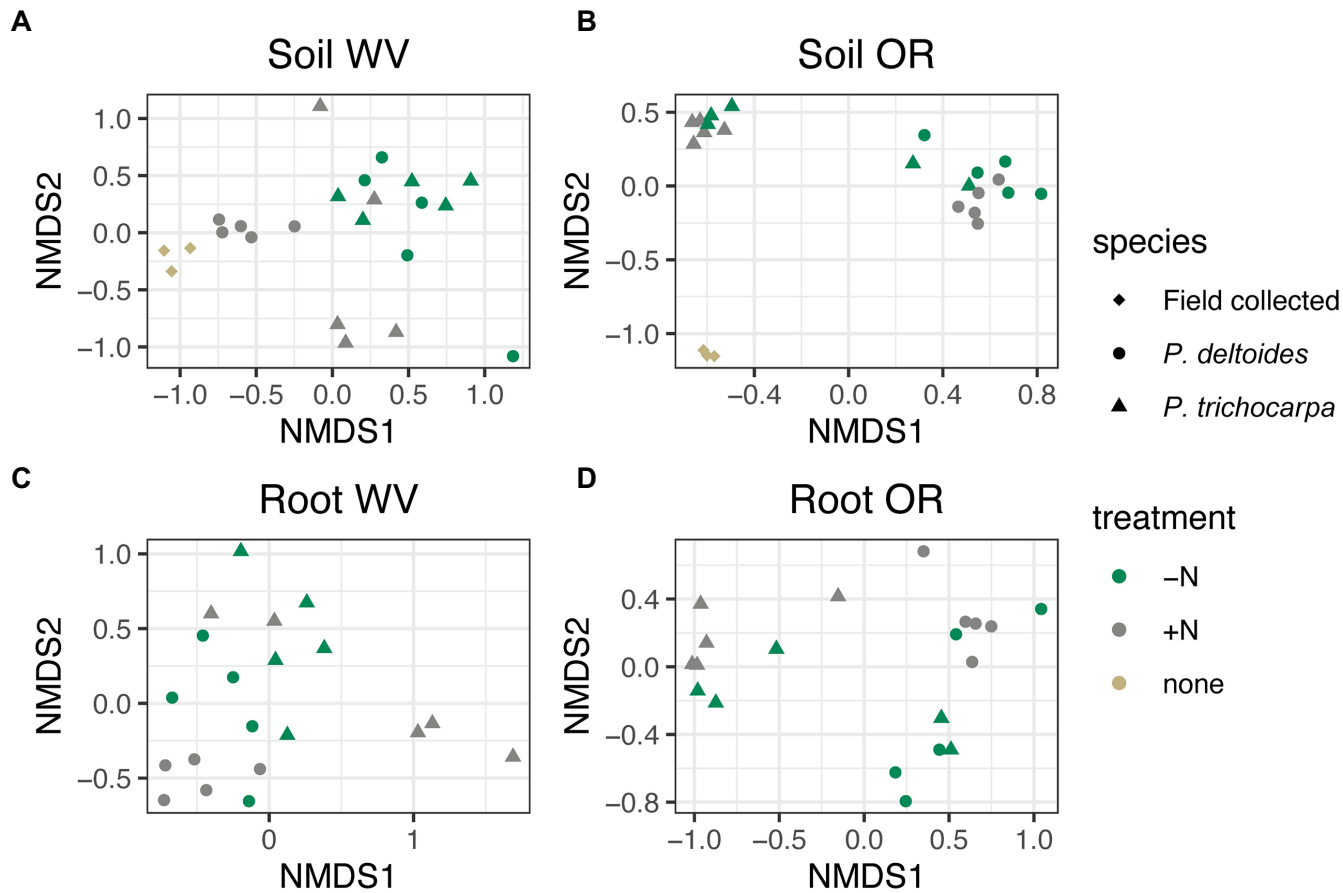
NMDS of Bray Curtis distance matrices for soil **(A,B)** and root **(C,D)** samples grown in soil collected from West Virginia (WV; **A,C**) or Oregon (OR; **B,D**). Each point corresponds to a sample, shape corresponds to tree species and color corresponds to nitrogen addition. Samples designated as “field collected” for species and “none” for treatment reflect starting soil communities collected from the field sites.

There was an interactive effect of tree species, soil origin, and N treatment on soil fungal community composition (Figure 2; R^2^ = 0.02, *p* < 0.001), but there was not an interaction between tree species, soil origin, and N treatment in roots (Figure 2; R^2^ = 0.02, *p* = 0.07). Soil origin was the largest factor impacting both soil and root fungal beta diversity explaining 30 and 35% of the variation in community composition, respectively (Supplementary Figure S2; *p* < 0.001) followed by tree species (R^2^ = 0.14 - soil and R^2^ = 0.09 - root, *p* < 0.001), and N treatment (R^2^ = 0.04 - soil and R^2^ = 0.03 root, *p* < 0.001).

### 3.2. Fungal core taxa

We identified one core taxa assigned to the genus *Rhizophagus* that accounted for 8.6% of reads in all root samples and 13 core taxa in all soil samples that accounted for 41.9% of reads and consisted of *Trichoderma*, *Tetracladium*, *Gibellulopsis*, *Metacordyceps*, *Inocybe*, *Metarhizium*, *Solicoccozyma*, *Talaromyces*, *Saitozyma*, and other taxa unable to be classified to the genus level (Supplementary Table S2). A single core fungal genus, the AM fungi *Rhizophagus*, occupied the roots of *P*. *deltoides* with the highest abundance when grown in soil collected from West Virginia (25.2% relative abundance) compared to *P*. *deltoides* roots grown in Oregon soil (0.6% relative abundance). *P*. *trichocarpa* root samples contained the single fungal core genus *Inocybe* that dominated the roots of *P*. *trichocarpa* grown in Oregon soils (52.4%) but decreased in relative abundance in roots of *P*. *trichocarpa* when grown in West Virginia soils (11.6%). Soil samples of *P*. *trichocarpa* and *P*. *deltoides* contained 10 and 7 core genera, respectively (Supplementary Table S3). Soil samples from both species were dominated by *Inocybe* (55.8% relative abundance) except for both *P*. *trichocarpa* and *P*. *deltoides* grown in +N West Virginia soils (8.2% relative abundance).

### 3.3. Response of arbuscular mycorrhizal and ectomycorrhizal fungi

**Figure 3:**
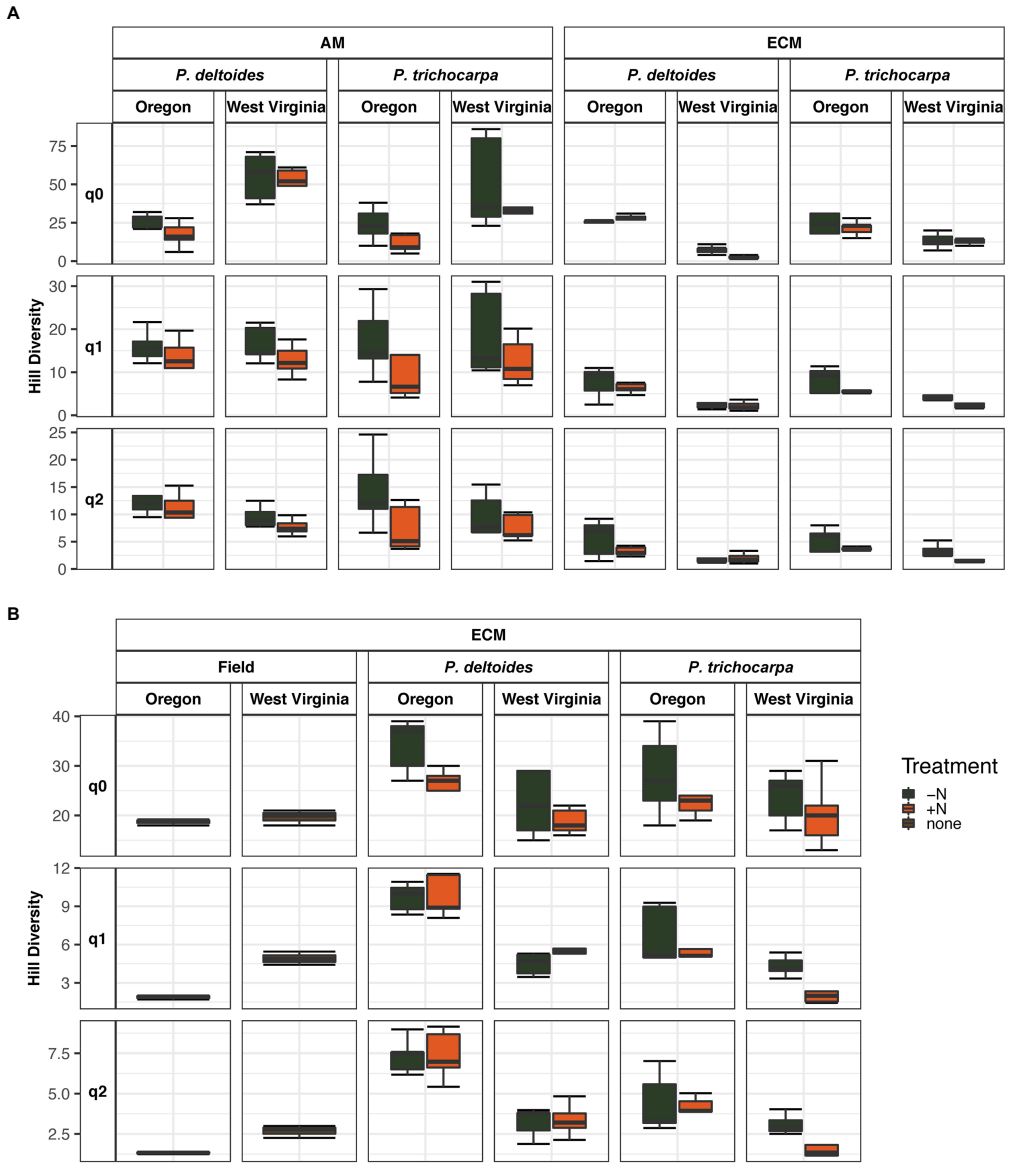
Boxplot of alpha diversity of FUNguild assigned arbuscular and ectomycorrhizal fungi measured by Hill numbers (q = 0, q = 1, q = 2) across nitrogen addition, tree species and soil origin. Lines within the boxplots represent median, 25th and 75th percentile values, while whiskers are defined by the largest value not greater than 1.5× the interquartile range (IQR) and the smallest value not less than 1.5× the IQR.

Soil origin strongly influenced AM and ECM fungal alpha diversity in roots and ECM alpha diversity in soil samples (*p* < 0.001 – Figure 3; Table 2). Nitrogen addition reduced ECM alpha diversity in the soil (Supplementary Table S4; *p* < 0.001) but not in the roots (*p* = 0.77). Ectomycorrhizal diversity was on average higher in *P*. *deltoides* than *P*. *trichocarpa* (Supplementary Tables S4, S5; *p* < 0.01). The addition of N decreased AM fungal relative abundance in *P*. *trichocarpa* roots compared to *P*. *deltoides* roots (Supplementary Table S6).

**Table 2:** Alpha diversity results from analysis of variance tests of the relationship between of FUNguild assigned arbuscular (AM) and ectomycorrhizal (ECM) fungal community Hill numbers (q = 0, q = 1, q = 2) and nitrogen addition, tree species and soil origin.

	ECM						AM					
Effects (df)	q = 0		q = 1		q = 2		q = 0		q = 1		q = 2	
	*F*	*P*	*F*	*P*	*F*	*P*	*F*	*P*	*F*	*P*	*F*	*P*
	*Soil*	
Nitrogen addition (1)	7.47185	**<0.01**	2.18032	0.15	1.06036	0.31						
Species (1)	0.35021	**0.01**	46.568	**<0.001**	32.1775	**<0.001**						
Soil origin (1)	12.0319	**<0.001**	61.2924	**<0.001**	51.4921	**<0.001**						
Species: nitrogen addition (1)	0.49632	0.49	7.38399	**0.01**	1.57955	0.22						
Soil origin: nitrogen addition (1)	0.57891	0.45	0.04141	0.84	0.31611	0.58						
Species soil origin (1)	2.0655	0.09	7.6672	**<0.001**	10.9604	**<0.001**						
Species: soil origin: nitrogen addition (1)	0.42009	0.52	0.79373	0.38	0.52268	0.47						
	*Root*	
Nitrogen addition (1)	0.08779	0.31	1.36953	**0.04**	5.37015	0.12	0.98088	0.18	0.81498	**0.03**	1.26449	**<0.01**
Species (1)	0.44444	0.51	0.1558	0.7	0.06673	0.8	0.06675	0.8	0.11368	0.74	0.33161	0.57
Soil origin (1)	48.4829	**<0.001**	16.008	**<0.01**	11.6051	0.06	9.4792	**<0.01**	0.01382	0.71	1.73784	0.08
Species: nitrogen addition (1)	0.88889	0.35	0.44239	0.51	0.24895	0.62	0.06698	0.8	0.8444	0.37	1.33107	0.26
Soil origin: nitrogen addition (1)	2.15089	0.38	0.26831	0.72	2.42896	0.89	0.14486	0.71	0.00128	0.68	0.07352	0.2
Species soil origin (1)	3.96159	0.06	0.78217	0.38	1.10677	0.3	0.02318	0.88	0.03235	0.86	0.10789	0.74
Species: soil origin: nitrogen addition (1)	2.77778	0.11	0.01153	0.92	0.99818	0.33	0.28972	0.59	0.10491	0.75	0.54286	0.47

Significant values (*p* < 0.05) are bolded. Within the soil, there were no classified AM fungi.

**Figure 4:**
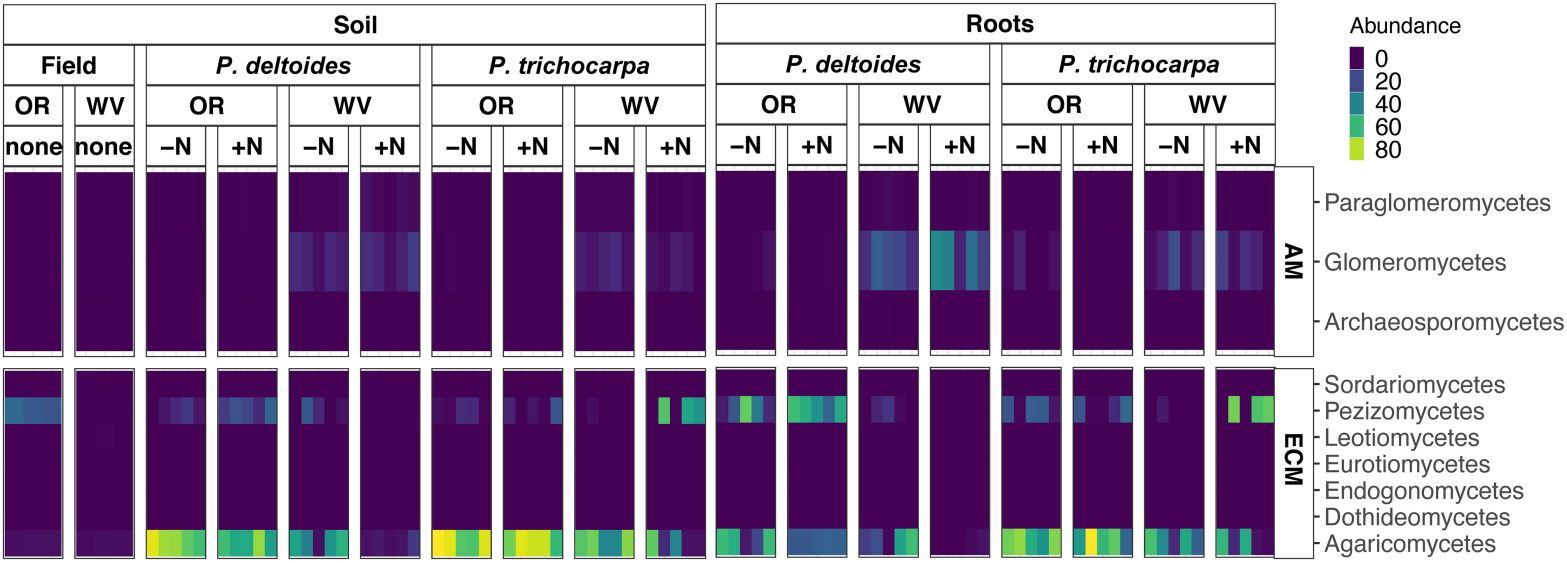
**(A)** Heatmap of FUNguild assigned arbuscular mycorrhizae (AM) and **(B)** ectomycorrhizae (ECM) fungal class relative abundance of *P*. *trichocarpa* and *P*. *deltoides* root and soil samples with nitrogen addition and different initial soils (WV, West Virginia; OR, Oregon).

The ECM fungal class Agaricomycetes dominated all root and soil samples of *P*. *trichocarpa* (Figure 2; 46.8% - soil, and 59.6% average relative abundance - root), but interestingly did not dominate the original soil samples collected from Oregon and West Virginia (Figure 4). The Agaricomycetes also dominated *P*. *deltoides* soils and roots (Figure 4; 57.2 and 32.3% average relative abundance respectively) except for *P*. *deltoides* when grown in its native West Virginia soil with N addition which resulted in a drastic decrease in this fungal class down to 1.3% root relative abundance and 6.3% soil relative abundance. When grown in W. Virginia soil, *P*. *deltoides* roots were instead dominated by the AM fungi Glomeromycetes (31.2% relative abundance) which is higher than all other samples (3.2% average relative abundance across other samples).

There was an interactive effect of soil origin, tree species, and N addition on ECM community composition in the soil (R^2^ = 0.04, *p* = 0.01). Root ECM and AM fungal beta diversity varied by soil origin (R^2^ = 0.60 - ECM and R^2^ = 0.31 - AM, *p* < 0.001) followed by tree species (R^2^ = 0.03 - ECM, R^2^ = 0.07 - AM, *p* < 0.05). The addition of N did not alter AM or ECM root fungal community composition (PERMANOVA, *p* > 0.05) but did alter ECM fungal composition in the soil (R^2^ = 0.04, *p* < 0.05).

### 3.4. Soil chemistry and plant characteristics correlation with taxa

Soil origin had only minor effects on plant growth characteristics, such as leaf number, leaf C:N, root %N, and stem %C (Supplementary Table S7). *P*. *trichocarpa* grew more rapidly than *P*. *deltoides* and had 10% higher leaf SPAD content, 50% greater height, 54% more leaves, and a higher C:N ratio in roots, leaves, and stems (*p* < 0.001; Table 3). Nitrate and ammonium were 80 and 29% higher in *P*. *deltoides* than *P*. *trichocarpa* soils at the end of the experiment (*p* < 0.001; Table 3). Nitrogen significantly increased plant growth rate for both species grown in both soil types. For instance, plant height was, on average, 133 and 111% greater for *P*. *trichocarpa* and *P*. *deltoides*, respectively, and leaf number was 193 and 49% greater for greater for *P*. *trichocarpa* and *P*. *deltoides*, respectively, when N was added (Table 3). Leaf number, stem %N, and soil nitrate varied by *Populus* species, soil origin, and N addition (*p* < 0.001; Table 3).

**Table 3:** Summary of soil and plant characteristics (mean ± standard error).

	*P*. *trichocarpa*				*P*. *deltoides*				Oregon soil origin		West Virginia soil origin		Oregon soil origin		West Virginia soil origin	
	−N	+N	−N	+N	−N	+N	−N	+N
Diameter	5.4 ± 0.45	8.1 ± 0.51	6.2 ± 0.74	7.7 ± 0.72	7.2 ± 1.09	9.8 ± 0.95	6.9 ± 0.99	9 ± 1.04
Height	39.8 ± 12.15	129.2 ± 7.76	56.1 ± 1.34	120.8 ± 7.05	36.4 ± 6.43	86.8 ± 3.4	36.8 ± 2.86	70.4 ± 3.65
Leaf number	16.8 ± 3.35	64.8 ± 16.51	20 ± 3.08	43 ± 13.03	18.2 ± 2.05	30 ± 2	18.4 ± 2.07	27.6 ± 3.85
SPAD	30.2 ± 5.8	34.8 ± 3.19	22.7 ± 1.57	33.2 ± 1.77	25.4 ± 2.03	28.1 ± 1.75	25.2 ± 1.56	30.7 ± 1.87
Leaf C:N	24.7 ± 2.95	20.2 ± 3.54	31.7 ± 1.59	20.3 ± 2.1	25.1 ± 0.97	20.7 ± 1.38	28.1 ± 3.08	19.7 ± 2.72
Leaf %C	46.5 ± 0.33	47.4 ± 0.81	47 ± 0.47	47.9 ± 0.84	46.1 ± 0.84	50.1 ± 0.61	47.3 ± 1.44	51.6 ± 0.77
Leaf %N	1.9 ± 0.21	2.4 ± 0.56	1.5 ± 0.07	2.4 ± 0.25	1.8 ± 0.09	2.4 ± 0.14	1.7 ± 0.17	2.7 ± 0.34
Root C:N	58.5 ± 16.01	38.4 ± 4.05	58.9 ± 10.24	39.8 ± 2.05	46.8 ± 2.1	43.6 ± 3.89	44.4 ± 6.25	40.9 ± 3.01
Root %C	42 ± 5.69	29 ± 4.27	38.5 ± 6.7	40.1 ± 6.02	44.7 ± 6	36.5 ± 4.89	42.5 ± 11.25	44.1 ± 5.3
Root %N	0.7 ± 0.12	0.8 ± 0.04	0.7 ± 0.09	1 ± 0.1	1 ± 0.12	0.8 ± 0.09	0.9 ± 0.2	1.1 ± 0.09
Stem C:N	132.6 ± 17.06	128.4 ± 28.97	154.3 ± 10.32	129.5 ± 13.03	96.5 ± 12.41	139.9 ± 30.94	113.4 ± 11.83	100.5 ± 12.46
Stem %C	48.2 ± 0.22	47.6 ± 0.79	48.5 ± 0.61	48.6 ± 1.31	46.6 ± 0.56	49.4 ± 0.78	46.9 ± 0.22	49.4 ± 0.34
Stem %N	0.4 ± 0.05	0.4 ± 0.09	0.3 ± 0.03	0.4 ± 0.03	0.5 ± 0.07	0.4 ± 0.06	0.4 ± 0.05	0.5 ± 0.06
Soil pH	5.3 ± 0.07	5.3 ± 0.09	5.5 ± 0.08	5.5 ± 0.06	5.2 ± 0.1	5.1 ± 0.04	5.6 ± 0.08	5.6 ± 0.08
Soil NH4	0.9 ± 0.2	0.9 ± 0.22	0.6 ± 0.09	0.9 ± 0.21	1.1 ± 0.24	1.4 ± 0.12	0.6 ± 0.1	1.1 ± 0.48
Soil NO3	1.4 ± 0.82	2.9 ± 0.88	1.6 ± 0.41	10.1 ± 2.02	2.7 ± 0.61	12.5 ± 2.99	2.3 ± 1.34	11.2 ± 2.74

**Figure 5:**
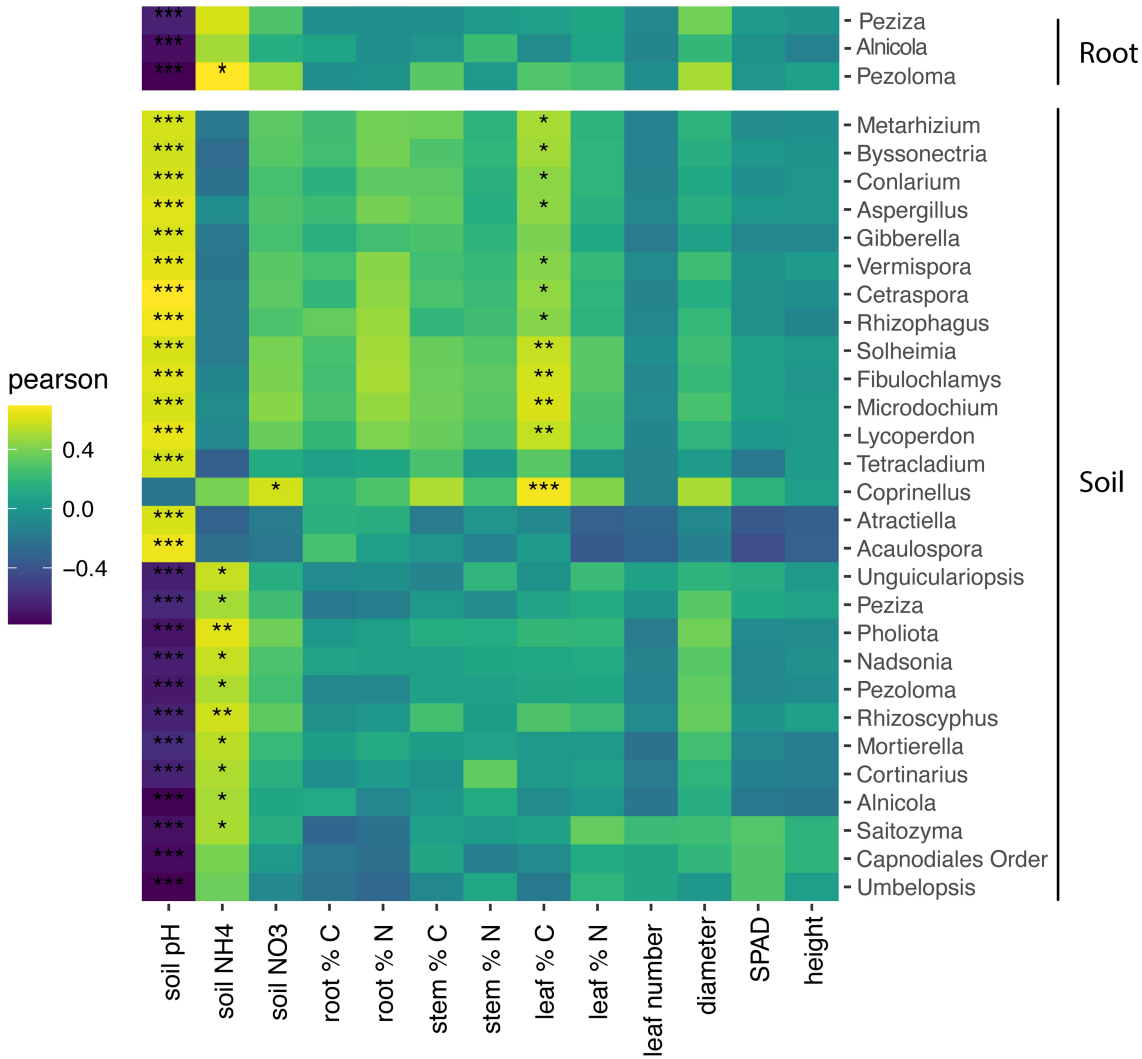
Heatmap of soil and root fungal genera correlations with soil and plant characteristics. Only taxa with significance in at least one environmental measurement is displayed. Pearson correlation test of the relationship between fungal genera and plant and soil characteristics with *p* values corrected for multiple comparisons by the false-discovery rate method. *P < 0.05; **P < 0.01; ***p < 0.001.

Within roots, the relative abundance three fungal genera, *Peziza*, *Alnicola*, and *Pezoloma*, negatively correlated with soil pH (*p* < 0.001) but did not significantly correlate with plant characteristics (Figure 5). Within the soil, the relative abundance of 11 fungal genera negatively correlated with soil pH, while the relative abundance of 13 fungal taxa positively correlated with soil pH (Figure 5; *p* < 0.05). Soil NH_4_ was positively correlated with the relative abundance of eight fungal taxa in the soil while the relative abundance of only one fungal taxon positively correlated to NO_3_ in the roots. The percentage of leaf carbon positively correlated to 12 fungal taxa in the soil (Figure 5; *p* < 0.05), but there were no other plant characteristics that correlated with fungal taxa in the roots.

## 4. Discussion

Management practices within ecosystems, like the addition of nutrients, have been shown to decrease beneficial symbiotic plant-fungal interactions (Wei et al., 2013; Johnson and Gibson, 2021). Consistent with this, we found that N addition significantly altered soil fungal communities, but the addition of N had a smaller impact on root endophytic fungal communities. Instead, within roots, we demonstrated that plant host and soil origin had a larger impact on fungal community diversity, composition, and taxa present, highlighting the dynamic nature of these interactions with implications for the functioning of these symbioses within both managed and natural ecosystems under global change.

Contrary to our hypothesis that N addition would result in convergence of the microbiome across plant species and soil types, we found that the addition of N had minimal impacts on the root mycobiome of *P*. *deltoides* and *P*. *trichocarpa*, but there were significant impacts of N addition on both soil fungal diversity and community composition. Fungal communities residing within plant roots are often well buffered to short term environmental perturbations (Gdanetz and Trail, 2017; Somenahally et al., 2018). Within roots, soil origin and tree species had the largest effect on fungal alpha diversity, community composition, and core taxa within this plant associated compartment. This result highlights the role plant selection may play on structuring the endospheric mycobiome, and how variation in fungal traits and life history strategy may influence their response to short term increases in soil N. Given the length of our experiment, it is possible that we have not reached a tipping point in soil N concentrations that will drive shifts in overall root fungal diversity and community composition (Lilleskov et al., 2019). Instead, this perturbation may impact distinct taxa present within roots and soils.

Core taxa are often hypothesized to have increased functional importance within the microbiome (Shade and Handelsman, 2012). Interestingly, the only core taxa we found in both *P*. *deltoides* and *P*. *trichocarpa* roots was the AM fungal taxa, *Rhizophagus*. Prior studies have demonstrated that *Populus* readily associate with *Rhizophagus* spp., and interactions with these fungi can influence plant nutrient uptake (Calabrese et al., 2019). Interestingly, we did not see an impact of N addition on the relative abundance of this core taxon within roots. Within our study, the addition of N profoundly accelerated plant growth relative to plants that were given no extra N. Nitrogen limitation in N− plants was evident by a greater C:N ratio in leaves and roots and lower chlorophyll density. Over the course of the greenhouse experiment, soils were generally depleted in NH_4_^+^ for both N+ and N− samples, with NH_4_^+^ being only slightly higher in N+ pots. Soil NO_3_^−^ was also lower in greenhouse endpoint samples relative to field soils, but the difference between N+ and N− was more remarkable than with NH_4_^+^ (Table 3). Thus, associations with *Rhizophagus* under both N treatments may reflect an overall nutrient limitation regardless of N treatment during this greenhouse study when plants were actively growing.

Nitrogen and phosphorus (P) are essential nutrients for plant growth. Within plant-mycorrhizal symbioses, plants provide C to the mycorrhizal community *via* root exudation and the mycorrhizal fungi directly exchange N and P with their plant hosts. Under N or P limitations, plants invest more C in mycorrhizal fungi (Treseder, 2004) because of the essential role these fungi contribute to nutrient uptake (Marschner and Dell, 1994; George et al., 1995). Conversely, if N or P availability increases, mycorrhizal-plant interactions often decline as the plants allocate carbohydrates elsewhere, thereby limiting C availability to soil fungi. In our study, N addition decreased overall mycorrhizal diversity and altered mycorrhizal fungal communities in the soil. Within the roots, we saw no effect of N addition on mycorrhizal diversity or relative abundance of core mycorrhizal taxa. We hypothesize that N addition did not affect C availability in roots, but available C in the soil may be altered. While there are clear changes in soil mycorrhizal communities with N addition, it is not clear if these shifts in mycorrhizal communities will lead to altered fungal community function or if these changes will persist if N levels return to ambient.

Mismatches between host plant species and soil inoculum may result in decreased host performance especially in response to abiotic stress. Within the soil, we identified 14 unique core taxa that accounted for ~42% of reads. Two of these taxa, *Trichoderma* (Guzmán-Guzmán et al., 2019) and *Inocybe* (Bonito et al., 2016) are known plant beneficial fungi. Within our soils, these taxa make up 23.4 and 55.8% relative abundance, respectively. Interestingly, the ECM fungi *Inocybe*, was a core taxon detected in *P*. *trichocarpa* roots. This taxon had a greater relative abundance within *P*. *trichocarpa* roots when they were grown in their native Oregon soil (52.4%) but decreased in relative abundance in roots of *P*. *trichocarpa* when grown in non-native West Virginia soils (11.6%). Similarly, *P*. *deltoides* roots had greater relative abundance of the AM fungi *Rhizophagus* when grown in its native W. Virginia soil relative to the non-native Oregon soil. Surprisingly, this mismatch of soil origin only had minor effects on plant growth, but as plants mature, these effects could become more pronounced. Future studies should examine the long-term outcomes of mismatches between plant host and soil fungal symbionts. Given the desire to grow *Populus* for pulp, paper, and biofuel, and the role climatic change may play in shifting the range of these trees in temperate forests, mismatches between plant hosts and symbiotic fungi could have long term consequences within both managed and natural ecosystems (Argüelles-Moyao and Garibay-Orijel, 2018; Peterson and Bode, 2021).

## 5. Conclusion

Overall, our results highlight the importance of plant species and soil origin on plant-fungal interactions. Nitrogen addition had large effects on fungal diversity, community composition, and the relative abundance and composition of symbiotic mycorrhizal fungal communities, but these effects were most pronounced within soils, not roots, and often contingent upon plant species and soil origin. Future endeavors should extend investigations to understand the long-term impacts of nutrient addition mediated shifts in fungal communities on plant establishment, plant growth and health, nutrient uptake, and subsequent changes in ecosystem function.

## Data availability statement

The datasets presented in this study can be found in online repositories. The names of the repository/repositories and accession number(s) can be found at: https://www.ncbi.nlm.nih.gov/, PRJNA875147.

## Author contributions

AAC and MAC contributed to the conception and design of the study and wrote the manuscript. DMK, ERJ, MAC, MMC, and SSJ conducted the study, measured plant phenotypes, and contributed to laboratory analyses. AAC, BBH, and ES provided statistical analyses for the manuscript. All authors contributed to the article and approved the submitted version.

## Funding

This research was funded as part of the Center for BioEnergy Innovation (CBI) at ORNL. CBI is a U.S. Department of Energy Bioenergy Research Center supported by the Office of Biological and Environmental Research in the DOE Office of Science. This research used resources of the Compute and Data Environment for Science (CADES) at the Oak Ridge National Laboratory, which is supported by the Office of Science of the U.S. Department of Energy under Contract No. DE-AC05-00OR22725. ES was supported in part by the U.S. Department of Energy, Office of Science, Office of Workforce Development for Teachers and Scientists (WDTS) under the Science Undergraduate Laboratory Internships program. BH was supported in part by the GEM Fellowship Program.

## Conflict of interest

The authors declare that the research was conducted in the absence of any commercial or financial relationships that could be construed as a potential conflict of interest.

## Publisher’s note

All claims expressed in this article are solely those of the authors and do not necessarily represent those of their affiliated organizations, or those of the publisher, the editors and the reviewers. Any product that may be evaluated in this article, or claim that may be made by its manufacturer, is not guaranteed or endorsed by the publisher.
