# Supplementary material for: Nitrogen addition alters soil fungal communities, but root fungal communities are resistant to change
Source: Front Microbiol. 2023 Jan 25;13:1033631. doi: 10.3389/fmicb.2022.1033631 (PMC9905728; doi:10.3389/fmicb.2022.1033631)
Supplement: Supplementary file 1 [file Data_Sheet_1.DOCX]

Supplementary Material

# Supplementary Figures and Tables

## Supplementary Figures

**Supplementary Figure 1**: NMDS of Bray Curtis distance matrices for soil collected from West Virginia or Oregon. Each point corresponds to a sample and color corresponds to field collection site.


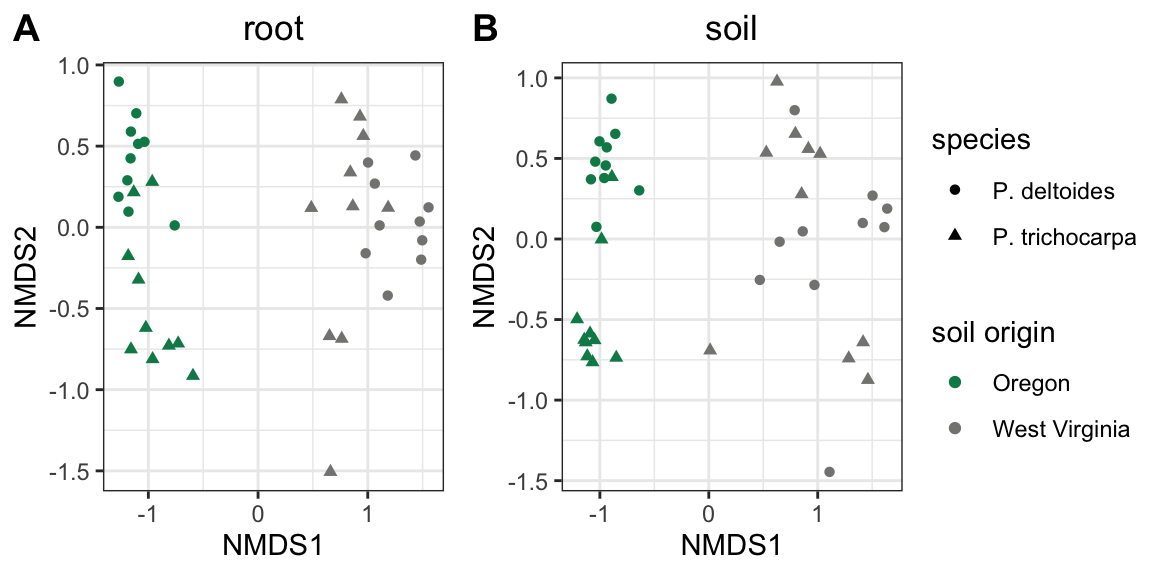


**Supplementary Figure 2**: NMDS of Bray Curtis distance matrices for root (A) and soil (B) samples grown in soil collected from West Virginia or Oregon. Each point corresponds to a sample, shape corresponds to tree species and color corresponds to soil origin.

## Supplementary Tables

**Supplementary Table 1**: Soil chemistry for soil samples collected from West Virginia and Oregon. Soils were then mixed with sand before utilization in the greenhouse experiment.

**Supplementary Table 2**: Core genera defined as genera in at least 95% of all soil or root samples.

**Supplementary Table 3:** Core genera defined as genera in at least 95% of *P. trichocarpa* or *P. deltoides* samples.

**Supplementary Table 4**: Tukey’s honest significant difference (HSD) *post hoc* analysis of soil arbuscular mycorrhizae (AM) fungal Hill diversity numbers (q = 0, q = 1, q = 2) and nitrogen addition, tree species and soil origin. Significant values (p < 0.05) are bolded, mean diff= mean difference, conf low= lower 95% confidence interval, conf high= upper 95% confidence interval.

**Supplementary Table 5**: Tukey’s honest significant difference (HSD) *post hoc* analysis of root ectomycorrhizae (ECM) fungal Hill diversity numbers (q = 0, q = 1, q = 2) and nitrogen addition, tree species and soil origin. Significant values (p < 0.05) are bolded, mean diff= mean difference, conf low= lower 95% confidence interval, conf high= upper 95% confidence interval.

**Supplementary Table 6:** Tukey’s honest significant difference (HSD) *post hoc* analysis of root arbuscular mycorrhizae (AM) fungal Hill diversity numbers (q = 0, q = 1, q = 2) and nitrogen addition, tree species and soil origin. Significant values (p < 0.05) are bolded, mean diff= mean difference, conf low= lower 95% confidence interval, conf high= upper 95% confidence interval.

**Supplementary Table 7**: General linear model (GLM) of plant and soil characteristics.
